# Evolution of technology convergence networks in Korea: Characteristics of temporal changes in R&D according to institution type

**DOI:** 10.1371/journal.pone.0192195

**Published:** 2018-02-08

**Authors:** Jae Young Choi, Seongkyoon Jeong, Jung-Kyu Jung

**Affiliations:** 1 Graduate School of Technology & Innovation Management, Hanyang University, Seoul, South Korea; 2 W.P. Carey School of Business, Arizona State University, Tempe, Arizona, United States of America; 3 Korea Institute of Science & Technology Evaluation and Planning (KISTEP), Seoul, South Korea; Beihang University, CHINA

## Abstract

This study investigates the temporal changes in development of technology convergence networks by institution type, *i*.*e*., public research institute (PRI), university and industry. Using the co-classification of technological domains of patents, we identified technology convergence of Korean patents, which were filed at Korea Intellectual Properties Office (KIPO) from 1997 to 2011. We conducted a network analysis at the technology level to search for the key technology fields and frequent instances of technology convergence. The results show that technology convergence networks have grown significantly in the recent period regardless of the institution type. While industries started to conspicuously engage in technology convergence in the late 1990s, universities or PRIs did not do so until the mid-2000s. This discrepancy in the phase of technology convergence is attributed to the temporal difference in R&D stage (*e*.*g*., basic research and commercial product development). Our findings imply that corporal and governmental R&D management decision on promising technology fields will be more effective if the decision makers carefully consider the type of R&D entity in analyzing technological landscapes.

## Introduction

Changes in technologies often influence the strategies of research and development (R&D) entities so significantly that a company, an industry, or even a country may win or lose a competitive edge [1]. Correspondingly, researchers in the field of innovation have observed interactions between such technological changes, paying significant attention to “convergence” [2–6].

As R&D entities have pursued strategic competitiveness of their products or technologies, the coverage of the term “convergence” has been expanded to industrial, sociological or even economical hybridization. Despite such widespread cognition, however, researchers do not seem to reach a consensus on the clear definition of the buzzword “convergence”. Still, a few notable studies have successfully founded the definitions and taxonomy of convergence. As a representative study, Curran and Leker [7] suggested that convergence be classified into “scientific convergence”, “market convergence”, “technology convergence” or “industry convergence.”

Since individual researchers or organizations in different technology fields tend to not collaborate together in general, technology convergence is regarded as one of the key solutions to socio-economic and technological problems in that it bridges the gap between distinct disciplines [5]. As a result, technology convergence can bring about not only technological and economic impact but also a cataclysmic impact on business activities. This is because technology convergence expands the current technological capability of incumbent R&D entities and influences the future directions of their R&D activities; moreover, even a transient phenomenon of technology convergence can cause a permanent change in the core strategies of an R&D entity.

Once recognizing the trend in technology convergence as a megatrend rather than dismissing it as just a fad, the R&D entity may desire to analyze and even forecast the trend to formulate an appropriate technology strategy and win against their competitors in the market, *e*.*g*., to secure a competitive edge in cost, quality and lead time of new product development. Implementation of such a strategy gives rise to radical changes in the business landscape. In the transient phase of technology convergence, even a newcomer can stir the established market, by taking over significant market share or even by creating a new industry [5].

In fact, technology convergence has been one of the core R&D strategies for a number of electronics, communication, energy, mechanics and biotechnology companies [8]. Universities and public R&D institutes (PRIs) have carried out a lot of technology convergence R&D activities as well. Given the importance of technology convergence, several developed countries including the United States, the European Union and Japan developed technology convergence roadmaps to coordinate their R&D activities to be more associated with technology convergence [9,10], expecting that technology convergence might lead to an increase in overlaps between the preexisting industries and that competition generated by such overlaps might result in industrial convergence. That is, the impact of technology convergence may in turn change diverse innovation-based activities such as types of service and products, industrial organizations and even governmental regulations and policies.

In formulating strategy for nourishing technology convergence, these national initiatives emphasize the role of different R&D entities. As a matter of fact, from the perspective of the innovation system, the roles of R&D entities have incessantly been revised to meet contemporary socio-economic needs [11]. In the “traditional” roles of R&D entities, universities are recommended to play a pioneering role in transferring the knowledge or technologies developed from basic researches to industries [12], and PRIs are recommended to fill the gap between universities conducting basic researches while industries develop and commercialize applications [13]. Industries, armed with the state-of-the-art instruments and equipment, persistently enhance in-house R&D capabilities and exploit the accomplishments of universities or PRIs. In other words, the challenge of industries, which is to bring and commercialize new disciplines to the market, is understood as contributing to the growth of national economy [14,15]. However, such stereotyped roles do not necessarily represent the characteristics of recent R&D activities, in which various overlaps and collaboration between different institution types occur [11,16–18].

Previous studies have qualitatively analyzed how different R&D entities contribute to technology convergence. However, most of them have offered limited scopes, in that they mostly base the findings on their case studies of specific technology fields, lacking holistic approaches (*i*.*e*., encompassing the full spectra of technologies). Such limited understandings of the technology convergence of the R&D subjects cannot fully meet the persistent needs for strategies to facilitate technology-convergent R&D. This necessitates researches on the overall quantitative trend of technology convergence according to the type of R&D entities.

The purpose of this study is to identify the characteristics of technology convergence of the three types of R&D entity: university, PRI and industry. We analyzed the trends in technology convergence according to the institution type using the patents filled to Korea Intellectual Property Office (KIPO) since the mid-1990s. Defining the patents classified into more than two technology fields as technology-convergent ones, we implemented the network analysis method to investigate the intersecting characteristics of the different technology fields into which the patents were classified [19–21].

The remnant of this paper is constructed as follows. In “Patent-Based Analyses of Technology Convergence,” previous studies on technology convergence are briefed. In “Data and Methods,” the measurements of the levels of technology convergence and the evolution characteristics in the networks are described. In “Characteristics of Technology Convergence in Korea by Institution Type” the results of the network analysis on technology convergence are followed by discussion on which type of institution played a central role in technology convergence. Differences in the role per institution type with the characteristics of the relevant technology fields are discussed as well. Finally, in “Conclusion,” the policy implications to facilitate technology convergence are summarized with the key findings of this study.

### Patent-based analyses of technology convergence

Considering the previous approaches that defined and measured technology convergence in the literature, we explain measurement of technology convergence using patents based on the network analysis.

#### Convergence, collaboration and networks

There are chances that R&D collaboration with external organizations brings forth development of technology convergence networks [5,22–24]. Because of limited capability and resources to satisfy market and industry demands, an R&D entity may implement collaborations with external organizations as a measure to hedge risks due to high cost and uncertainty of success [25]. Acceleration in evolution of technologies and intensification of, especially business, competition may lead to stronger demands for collaboration.

There have been various research questions regarding R&D collaboration networks, but most of them appear to be concentrated on academic [26], research-institutional [27], or collaboration among various type of institution [28]. Meanwhile, a number of corporal collaboration network analyses have been conducted in terms of R&D performance. For example, seemingly conflicting reports have been available suggesting an optimal network structure of alliance in which the performance, *e*.*g*. the rate of innovation is leveraged by appropriate combination of network density and heterogeneity [29] or a relatively loose structure [30,31] itself.

However, it is noted that those previous studies have focused on the characterization of the network structure in terms of maximizing the performance of innovation. On the contrary, scarce attention appears to have been paid to institution-type-specific preferences for activities of technology convergence. From the policy point of view, such a research question may also have practical significance if the temporal development of technology convergence can be identified.

#### Analysis of technology convergence using patents

Despite the ambiguous and multivalent nature of the term, a number of recent empirical researches appear to adopt essentially the same definition of technology convergence [7,32–38].

A number of quantitative studies on the mechanism of technology convergence used patent data [2,7,39] as the prime data objectively representing technology innovation [40–42]. This is because, having been scrupulously reviewed and confirmed by patent examiners with professional expertise, a patent is considered to contain authentic and detailed technological information. Examples of use of patents include the macroscopic trend analysis of technological changes [43] and the measurement of technology convergence [36].

Moreover, every patent is codified into classifications according to a systematic scheme of technology. This has a significant advantage [44,45] in that the patent classification provides a criterion of technology convergence. Several studies implemented the co-classification approach, in which a technology-convergent patent is defined as being simultaneously coded into more than two classifications [32,35,46]. Similar approaches to the co-classification have been used by studies on scientific convergence based on the subject categories presented by Web of Science to classify journals [47–49] and that on industrial convergence based on the standard industry codes [5,46], respectively.

We used the international patent classification (IPC) brought by World Intellectual Property Organization [50] to classify a patent into the technology fields. Since its introduction in 1971 based on the Strasbourg Agreement, IPC has been revised periodically to reflect technological trends. This study follows its most recent revision, IPC Rev. 8. Since Curran and Leker [7], the co-classification approach based on IPCs has been used to investigate technology convergence with variations in the configurations of classification [6,35]. Curran and Leker [7] and Geum *et al*. [35] investigated technology convergence between technologies related with phytosterols and between information technology (IT) and biotechnology with a focus on smartphone applications, respectively. Meanwhile, rather than focusing on a specified set of technology fields, Jeong *et al*. [6] performed a holistic analysis to include *all* the available fields of technology for the patents applied to Korean Intellectual Property Office (KIPO).

#### Previous studies on technology convergence networks

Despite the variety in the details such as index design and visualization methods, the network analysis method has been one of the key instruments of the technology convergence studies [51–54]. Originally devised by Barnes [55] to investigate human relationships, the method defines the structure of a network as a function of the participants and their relationships and investigates the network using graphs, linear algebra, statistical probabilities, simulations and so on [56–57].

Table 1 summarizes several previous studies of technology convergence based on the network analysis. Their use of a patent database confined to a local or national intellectual property office (IPO) seems to minimize redundancy in patent applications to multiple IPOs; if technology convergence is measured regardless of IPO, because of the territorial principle [40] in intellectual property (IP), the same convergence of technologies is counted in an overlapped way as the same patent is applied to a number of IPOs, causing inflated statistics of technology convergence.

**Table 1:** Overview of the previous literature on technology convergence that implemented network analyses.

Authors	Database	Technology classification using	Analysis method
Schoen *et al*. [51]	The Corporate Invention Board (CIB)	WIPO Technology Concordance Table	Co-classification
Leydesdorff *et al*. [58]	US Patents (USPTO)	IPC Classes and Subclasses	Cosine distance
Boyack and Klavans [53]	US Patents (USPTO)	IPC Subclasses	Co-classification
Kay *et al*. [54]	European Patents (EPO)	IPC Subclasses	Cosine distance

In a network analysis, a network is constructed based on the definition of the nodes and links and the structural characteristics of the network are investigated [59]. With technology fields [51] or IPCs [53] as nodes, the number of patents that can be co-classified to different technology fields [51] or IPCs [53] or the cosine distance [52,54] between the classification of a patent and that of cited patents represents the link. For example, Boyack and Klavans [53] constructed a patent network of which nodes and links corresponded to the IPC and co-classifications, respectively, and combined it with the author information from scientific journals to find which fields of science (from journals) and industry (from patents) were correlated stronger than other fields; Leydesdorff *et al*. [58] implemented the network analysis to identify the scientific impact of academic journals and convergent fields of technology, *e*.*g*., nanoscience, in which cosine distance was used to normalize both the cited- and citing journal networks.

## Data and methods

We define technology convergence as a crossover of different fields of technology. Such a definition of intersecting nature indicates that the network analysis method is an effective approach to characterize technology convergence [6,51,53]. The subjects of technology convergence in our study are the 35 fields of technology defined in the IPC-Technology Concordance Table by WIPO [50], which are in turn categorized into 5 technology sectors. Regarding a patent as the unit of innovation, we mapped each patent onto the fields of technology [6,51] according to the concordance table [60]. The current IPC system is not free from discrepancy between the classifications defined by IPC and other technological or industrial classifications of the actual products and services. This is why WIPO provides the IPC–Technology Concordance Table as a separate set of technology classification table. Consequently, a weighted network can be constructed, composed of nodes and links corresponding to the technology fields and occurrences of technology convergence, respectively. While the number of patent applications is considered as the size of a node, a link is weighted by the number of technology convergent patents, *i*.*e*. the number of crossovers between different technology fields.

To investigate the development of technology convergence characteristic of institution type, we built separate technology networks for PRIs, universities and industries, respectively. Then we performed network analyses on technology convergence for the 35 technology fields according to R&D entity type (either of university, PRI or industry) and time. We did not only characterize the evolution of technology convergence based on the changes of the networks in the number of links, density, etc. but also identified the key technology fields around which technology convergence occurred frequently. The time span of the patent applications from 1997 to 2011 was split into the three periods, *i*.*e*. from 1997 to 2001, from 2002 to 2006 and from 2007 to 2011, respectively.

A node and link can be described in Eqs (1) and (2), respectively, as

$$Node_{i}={\left(\mathrm{WIPO}\;\mathrm{technology}\;\mathrm{field}\right)}_{i}\;\mathrm{and}$$ (1)

$$Link_{ij}=(\mathrm{intersection}\;\mathrm{of}\;\mathrm{technology}\;\mathrm{fields}\;\mathrm{between}Node_{i}\mathrm{and}\;Node_{j}).$$ (2)

Our criterion of technology convergence as a crossover of technology fields is essentially similar with IPC co-classification, which in turn enables objective determination whether a patent is technology-convergent or not. It is because a classification is a part of the bibliometric information of a patent granted by examiners of a patent office with objective and technological considerations and thus no further modification or processing is required. A number of the previous researches considered technology convergence as crossover between different classification codes, *e*.*g*. IPCs. This imposes a problem because a patent classification code itself is not identical to a technology. It should be noted that a patent classification system such as IPC is literally for the convenience’s sake of patent examiners, *i*.*e*. in indexing patents.

In this study the weight of *Link*_*ij*_, *i*.*e*. *w*_*ij*_ is defined as

$$w_{ij}=(\mathrm{the}\;\mathrm{number}\;\mathrm{of}\;\mathrm{occurrences}\;\mathrm{of}Link_{ij}\;\mathrm{in}\;\mathrm{the}\;\mathrm{given}\;\mathrm{data}\;\mathrm{pool}),$$ (3)

which is equivalent to the number of patent applications belong to both technology fields *i* and *j*.

We performed both the network and node analyses. While the former characterizes the technology convergence indigenous to institution type, the latter locates the fields of technology onto which technology convergence was focused. For the node analysis, we defined and investigated *technological closeness* between fields of technology. The technological closeness is based on the idea that more similar technologies have higher chance of convergence [44]. We measured technological closeness between *Node*_*i*_ and *Node*_*j*_ in the same fashion that of Jaccard similarity [61]: denoting *N*(*i*) as the number of patent applications corresponding to *Node*_*i*_, the technological closeness *C*_*ij*_ is defined as

$$C_{ij}=\frac{N(i\cap j)}{N(i\cup j)}=\frac{w_{ij}}{N(i)+N(j)-w_{ij}}.$$ (4)

To characterize the technology convergence network as a whole, we introduced typical measures of network analysis. As a descriptive indicator of how much convergence prevails in a technology network, we adopted network density [62]. Network density is defined as the relative fraction of the number of actually observed links to the total number of possible links, corresponds to the diversity of technology convergence. For a network *n*, the density is defined as

$$Density_{n}=\frac{\sum_{i=1}^{35}\sum_{j=1}^{35}\{\begin{array}{c} 1|w_{ij}\neq 0\\ 0|w_{ij}=0 \end{array}}{{}_{35}\prod_{2}}.$$ (5)

To count the frequency of technology convergence for a technology field, average number of links was used. Average number of links stands for the number of occurrences of technology convergence for a given technology field and is defined in Eq (6) as

$$Average\;link_{n}=\frac{\sum_{i=1}^{35}\sum_{j=1}^{35}\{\begin{array}{c} 1|w_{ij}\neq 0\\ 0|w_{ij}=0 \end{array}}{35}.$$ (6)

In other words, average link number means literally how many fields of technology participated in technology convergence. Since we have defined the three periods, the average numbers of links in this study may be equivalent to the frequency of technology convergence.

To measure the chance of technology convergence that a technology field has, node strength was introduced. Strength of a node is defined as the sum of the weights from *Node*_*i*_ to the other nodes [63]. Essentially equivalent to node strength [64], Eq (7) suggests that the node strength increases as technology convergence generates more links in the given technology field. Note no use of combination as the diagonal matrix *Link*_ij_ is included in Eq (7).

$$Strength\;of\;Node_{i}=\frac{\sum_{j=1}^{35}w_{ij}-w_{ii}}{\sum_{j=1}^{35}\{\begin{array}{l} 1|w_{ij}\neq 0\\ 0|w_{ij}=0^{-1} \end{array}}$$ (7)

We used Cyram NetMiner 4 and Gephi 0.9 for network analysis and visualization, respectively. We used the Circle Pack layout to highlight linkages between technology sectors in which nodes or technology fields in the same technology sector are positioned in a rather closely clustered way, while those in different sectors are remotely scattered. The size of the nodes corresponds to the number of patent applications.

### Trends in technology convergence patent applications

**Fig 1:**
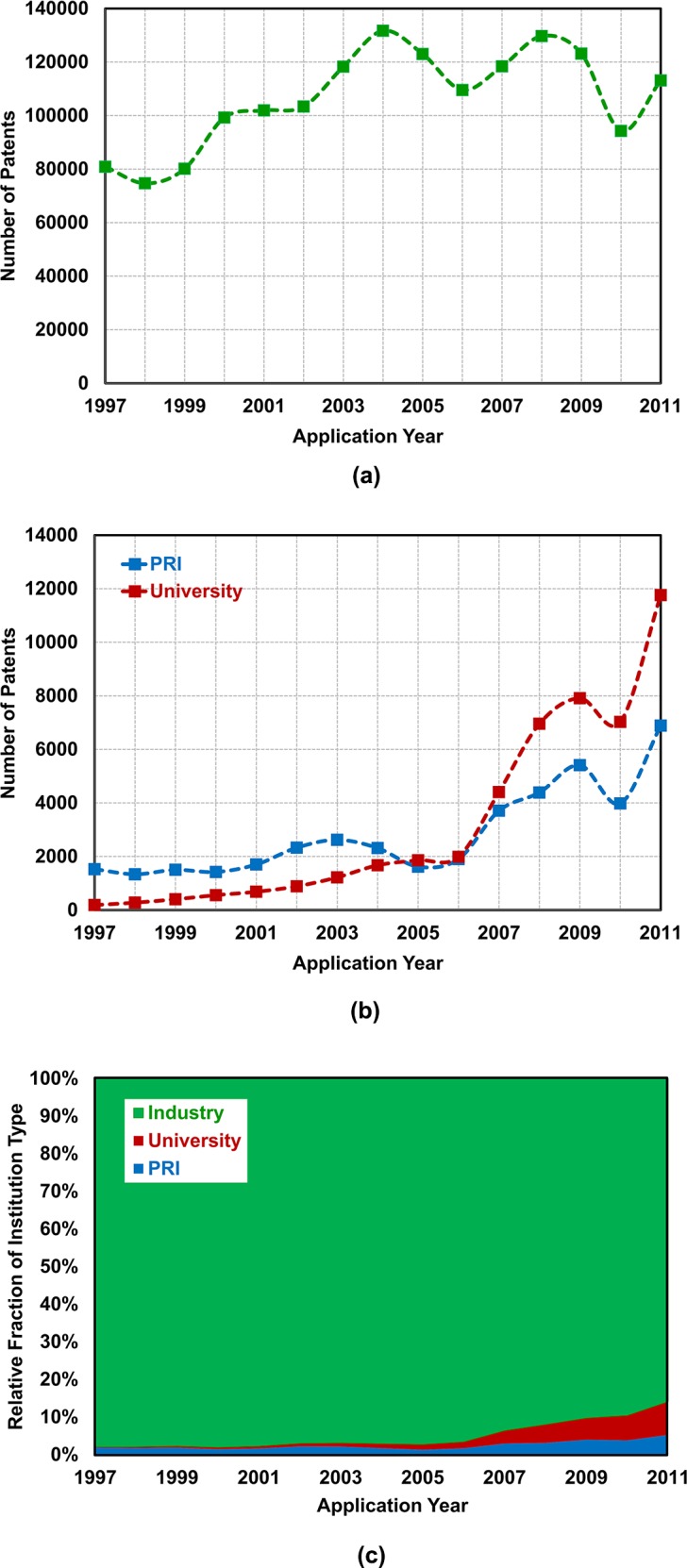
Changes in (a) the number of technology convergence patents filed by industries and (b) the numbers of technology convergence patents filed by public research institutes (PRIs) and universities with regard to application year. The relative fractions (*i*.*e*., the ratio of the number of such patents by institution type to the total number of patent applications per annum) of PRI-, university- and industry-filed technology convergence patents with regard to application year is shown in (c).

We performed a network analysis of technology convergence patents filed to KIPO with regard to the three periods from 1997 to 2011. To find whether different types of institution (university, PRI and industry) had characteristic trends of technology convergence patents, the patents were grouped according to the institution type of applicants. Changes in the numbers and relative fractions of patents by institution type are plotted in Fig 1(A) and 1(B), respectively, as a function of application year.

Among the three institution types, industries dominate the number of patents: 80,875 and 113,102 patents were applied to KIPO in 1997 and 2011, respectively. However, their increase rates were relatively lower than those of the other institution types. While the increase rate of industry patents was no more than 40 percent from 1997 to 2011, that of PRI patents was about 351 percent, from 1,526 applications in 1997 to 6,887 in 2011; that of university patents was even higher, that is, about 6,223 percent, from 186 applications in 1997 to 11,761 in 2011. Fig 1(A) also visualizes such differences in the increase rates by institution type. Explanations for the huge increase in university patents seem twofold. Firstly, universities have recently been in great vigor to collaborate R&D with industries, *e*.*g*., for technology commercialization [32,65]; secondly, universities have adopted the number of patent applications as one of the key indices in evaluating R&D performances of researchers [66,67].

The relative fractions of patent applications by institution type have characteristics essentially similar to that of the patent numbers. Again, industry patents predominate, occupying not less than 90 percent of the technology convergence patents. On the contrary, the relative fraction of university or PRI patents is relatively low, not more than 10 percent in 2011. It was not until the late 2000s that universities and PRIs began to increase their weight in the national innovation system of Korea [67]. For instance, there had been only a scarce increase, *e*.*g*. no less than 2 percent in 1997 as shown in Fig 1(B).

## Results and discussion

### Characteristics of technology convergence in Korea according to institution type

#### Evolution characteristics of technology convergence networks

Following Jeong *et al*. [6], we analyzed technology convergence networks of each institution type for Period 1 (1997–2001), Period 2 (2002–2006) and Period 3 (2007–2011). Table 2 summarizes the network analysis results by institution type and period, showing increase in both the density and average number of links. This is in accordance with the evolution characteristics shown in Fig 1.

**Table 2:** Characteristics of technology convergence networks by institution type.

Institution	Period	Number of links	Density	Average degree
PRI	1997–2001	261	0.19	6.457
	2002–2006	395	0.303	10.286
	2007–2011	929	0.751	25.543
University	1997–2001	159	0.104	3.543
	2002–2006	367	0.279	9.486
	2007–2011	1,031	0.837	28.457
Industry	1997–2001	937	0.758	25.771
	2002–2006	1,113	0.906	30.8
	2007–2011	1,175	0.958	32.571

Increase in the network density and average number of links of the PRI technology convergence networks from 0.190 in Period 1 to 0.751 in Period 3 and from 6.457 in Period 1 to 25.543 in Period 3, respectively, indicates convergence among technology fields. The technology convergence networks of universities have higher network density and average number of links than that of PRIs: the network density increased from 0.104 in Period 1 to 0.837 in Period 3, while the average number of links from 3.543 in Period 1 to 28.457 in Period 3. On the other hand, evolution of technology convergence networks in industries was larger in scale but slower in growth than that in universities or PRIs: the network density and average number of links increased from 0.758 in Period 1 to 0.958 in Period 3 and from 22,771 in Period 1 to 32,571 in Period 3, respectively.

Explanations for such high increase rates in university or PRI technology convergence appear twofold. Firstly, recently universities and PRIs have played a central role of collaborative R&D activities for technology convergence in Korea [65,67,68]. Secondly, the R&D of a university or PRI is often relatively free than the counterpart of an industry. Between them, universities rather than PRI are likely to conduct more freely R&D of convergent technologies [65,67]. The numbers described above agree with these explanations.

**Fig 2:**
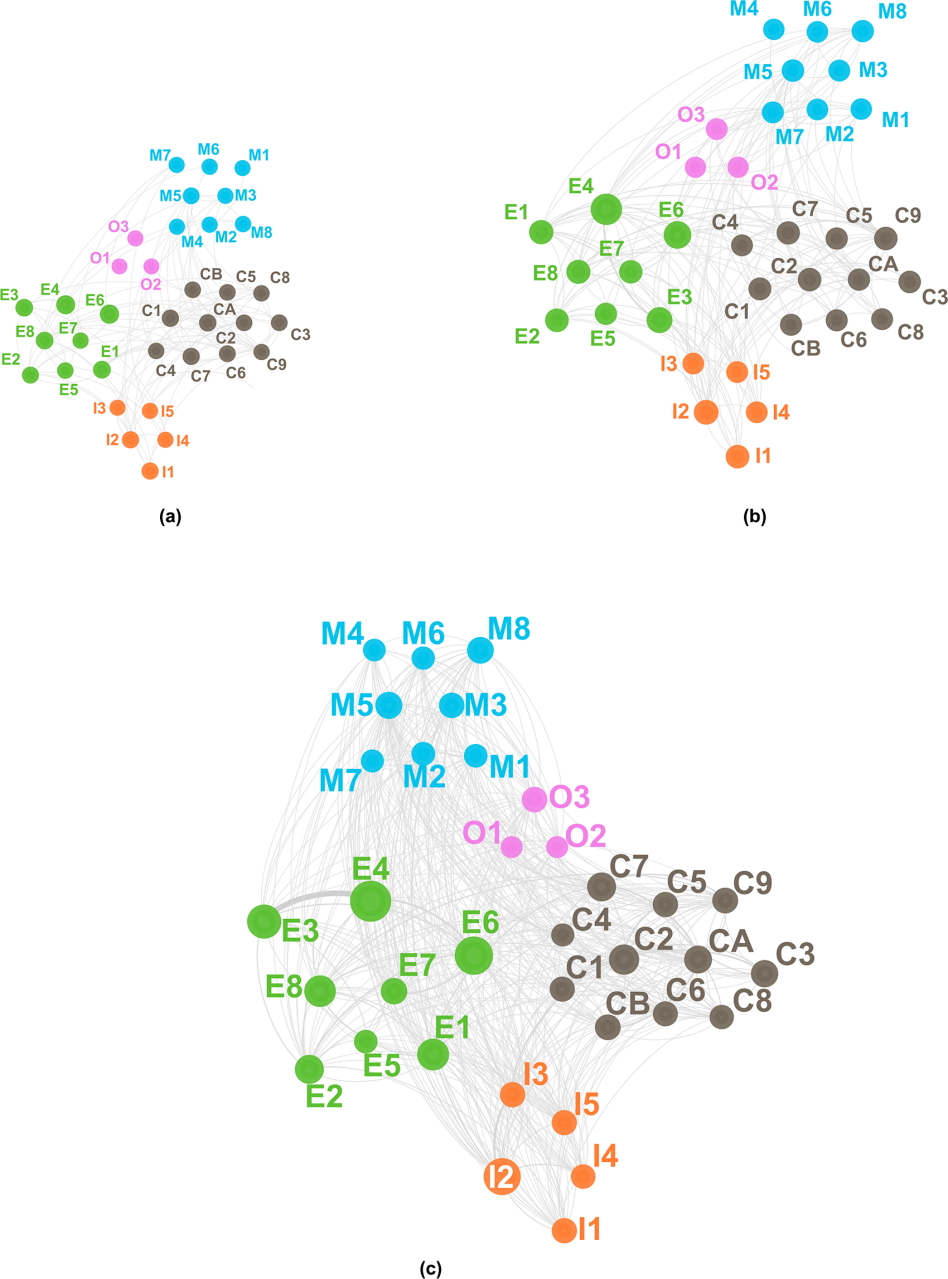
Technology convergence network of PRIs. (a) from 1997 to 2001, (b) from 2002 to 2006 and (c) from 2007 to 2011. Consult Table 3 for the symbols representing the technology fields. The node size is proportional to the number of patent applications corresponding to the field of technology; however, it should be noted that each node has the minimal size for the sake of visibility.

**Fig 3:**
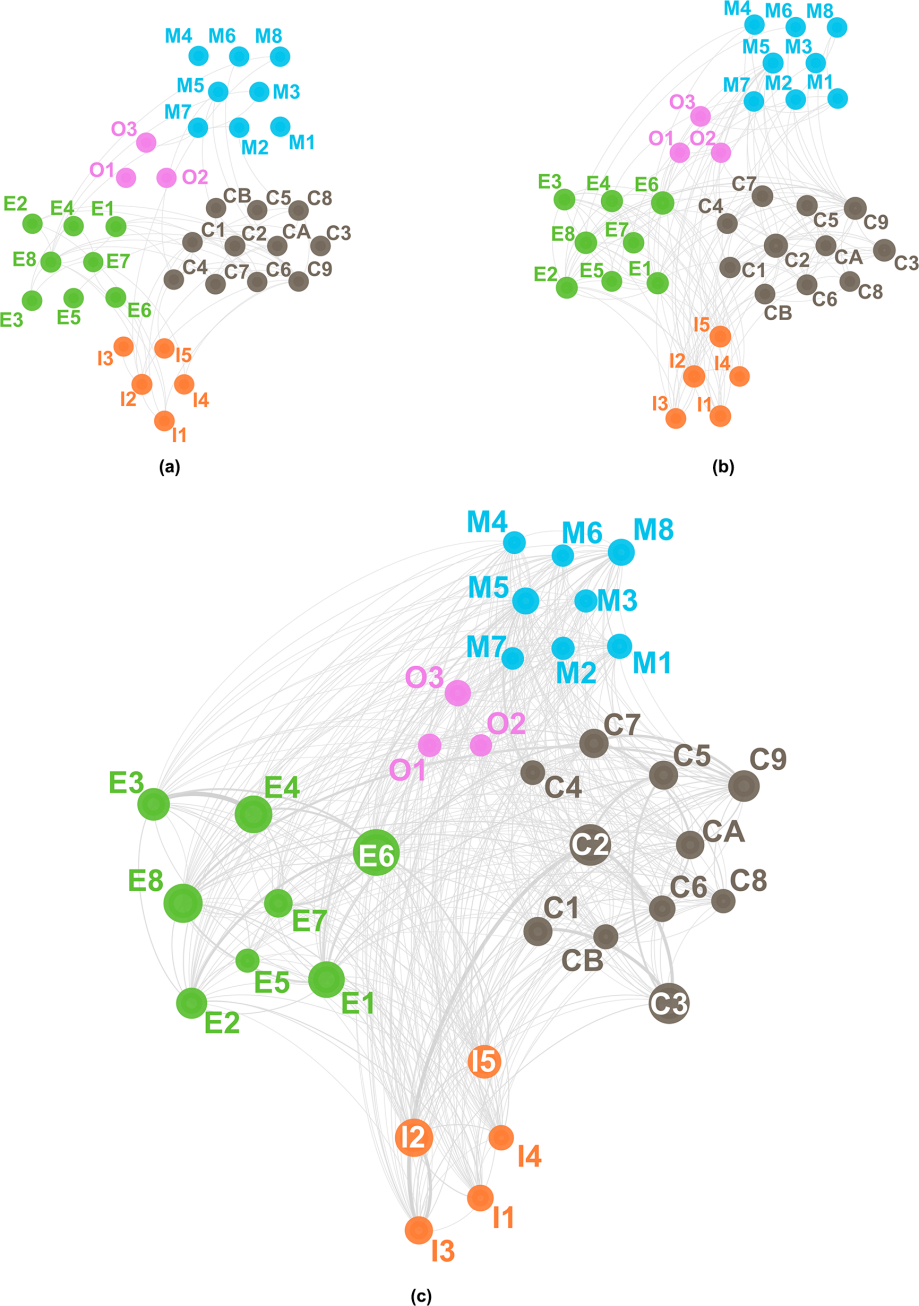
Technology convergence network of universities. (a) from 1997 to 2001, (b) from 2002 to 2006 and (c) from 2007 to 2011. Consult Table 3 for the symbols representing the technology fields. The node size is proportional to the number of patent applications corresponding to the field of technology; however, it should be noted that each node has the minimal size for the sake of visibility.

**Fig 4:**
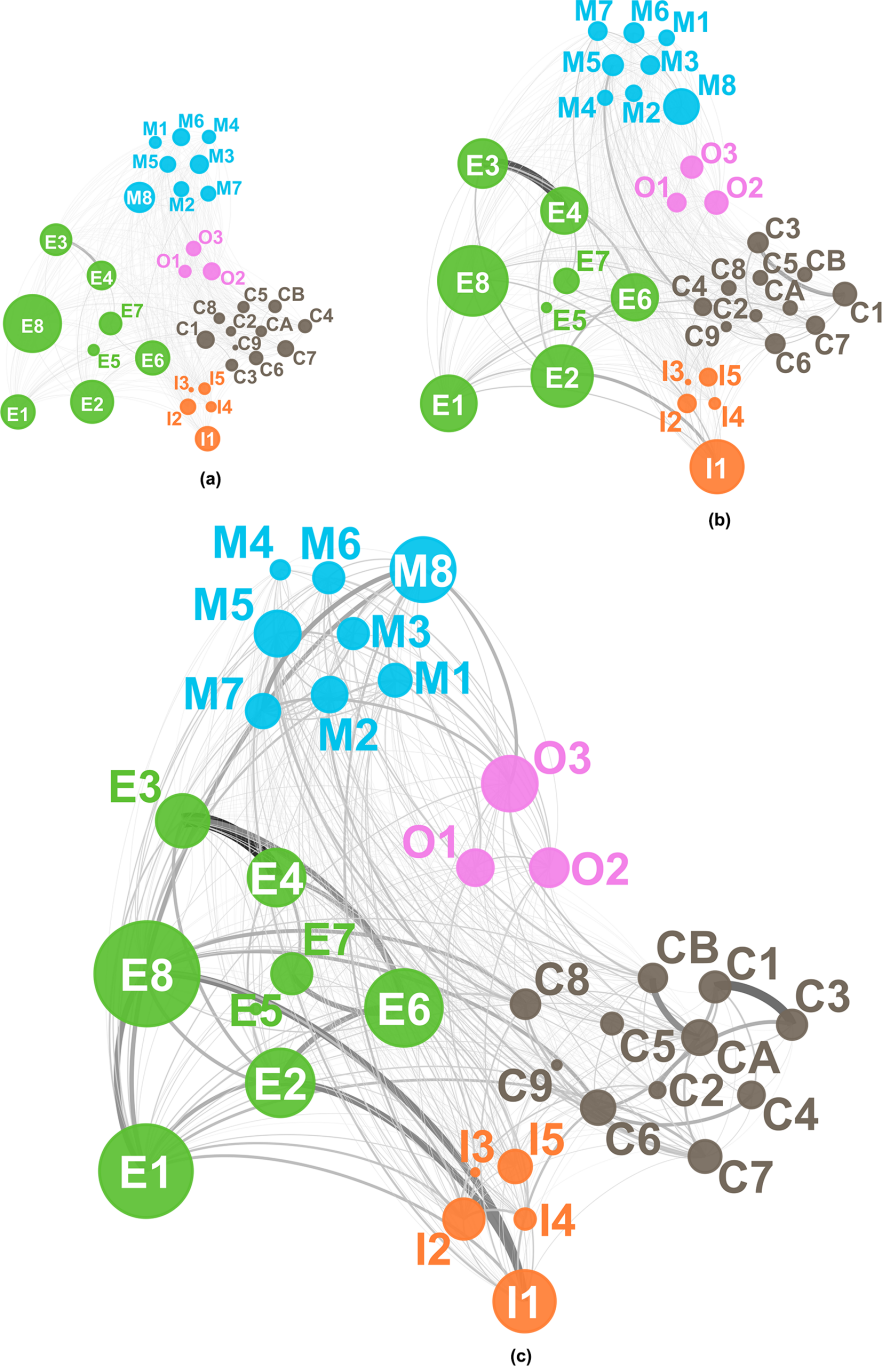
Technology convergence network of industries. (a) from 1997 to 2001, (b) from 2002 to 2006 and (c) from 2007 to 2011. Consult Table 3 for the symbols representing the technology fields. The node size is proportional to the number of patent applications corresponding to the field of technology; however, it should be noted that each node has the minimal size for the sake of visibility.

Figs 2, 3 and 4 visualize the evolution of technology convergence networks in universities, PRIs and industries, respectively. In each figure, (a), (b) and (c) correspond to Periods 1, 2 and 3, respectively. In Figs 2 to 4 we used abbreviated names of the technology fields in the *IPC and Technology Concordance Table* [50] for the sake of readability. The details of abbreviation are listed in Table 3.

**Table 3:** Description of symbols used in Figs 2, 3 and 4 corresponding to the technology sectors and fields given in the IPC-Technology Concordance Table (WIPO, 2012).

No.	Sector	Field	Symbol
1	Electrical engineering	Electrical machinery, apparatus, energy	E1
2		Audio-visual technology	E2
3		Telecommunications	E3
4		Digital communication	E4
5		Basic communication processes	E5
6		Computer technology	E6
7		IT methods for management	E7
8		Semiconductors	E8
9	Instruments	Optics	I1
10		Measurement	I2
11		Analysis of biological materials	I3
12		Control	I4
13		Medical technology	I5
14	Chemistry	Organic fine chemistry	C1
15		Biotechnology	C2
16		Pharmaceuticals	C3
17		Macromolecular chemistry, polymers	C4
18		Food chemistry	C5
19		Basic materials chemistry	C6
20		Materials, metallurgy	C7
21		Surface technology, coating	C8
22		Micro-structural and nano-technology	C9
23		Chemical engineering	CA
24		Environmental technology	CB
25	Mechanical engineering	Handling	M1
26		Machine tools	M2
27		Engines, pumps, turbines	M3
28		Textile and paper machines	M4
29		Other special machines	M5
30		Thermal processes and apparatus	M6
31		Mechanical elements	M7
32		Transport	M8
33	Other fields	Furniture, games	O1
34		Other consumer goods	O2
35		Civil engineering	O3

Fig 2 shows the evolution of technology convergence networks in the PRI patents. In Period 1, as depicted in Fig 2(A), very few incidences of technology convergence around the Electrical engineering sector, *e*.*g*., Telecommunications (E3) and Digital communication (E4), exist. Fig 2(B) shows the growth of technology convergence networks, especially among different sectors include Materials, metallurgy (C7), Micro-structural and nano-technology (C9) and Chemical engineering (CA) converging with Semiconductors (E8) and Measurement (I2). Such characteristics of convergence in Period 2 may be due to patent applications resulting from the governmental R&D investment in the nanoscience and nanotechnologies that began around the year 2000 [69]. Period 2 also shows an increase in IT convergence, *e*.*g*., Telecommunications (E3), Digital communication (E4) and IT methods for management (E7) with technologies of other sectors, *e*.*g*., Mechanical engineering. In Period 3, technology convergence networks prevail in most technology fields. For example, technologies of Instruments sector, mainly Measurement (I2), converged with technologies from other sectors such as Digital communication (E4), Semiconductors (E8), Transport (M8) and Micro-structural and nano-technology (C9), as seen in Fig 2(C).

Fig 3 visualizes the evolution of technology convergence networks in university patents. In Period 1, effectively no technology convergence seems available. Fig 3(B) shows early development of technology convergence networks in Period 2 across different technology sectors, *e*.*g*., between Micro-structural and nano-technology (C9) and Semiconductors (E8), and within the same sector of technology such as Materials, metallurgy (C7) and Micro-structural and nano-technology (C9). Here Micro-structural and nano-technology (C9) played a hub role in technology convergence. This is because the miniaturization of semiconductor devices was critical, of which addressment required interconnecting R&D activities in semiconductor devices, materials and processing technologies [70,71]. Other technologies developed their own convergence networks in Fig 3(B) as well. Correspondingly, Period 3 shows the explosive growth in technology convergence networks. Fig 3(C) highlights the Instruments sector technologies as the center of technology convergence with Chemistry sector technologies, *e*.*g*., Materials, metallurgy (C7) or Electrical engineering counterparts such as Digital communication (E4) and Computer technology (E6).

Fig 4 visualizes the evolution of the technology convergence networks in industries. Contrary to universities and PRIs, technology convergence networks in industries had appeared in Period 1. Fig 4(A) shows the networks between technologies of different sectors, *e*.*g*., Electrical engineering and Chemistry; Mechanical elements (M7) and Computer technology (E6) had networks as well. Furthermore, we find convergence in the same technology sector, particularly around either of the Electrical engineering or Chemistry sector. Instances include Digital communication (E4) and IT methods for management (E7), Telecommunications (E3) and Digital communication (E4), Organic fine chemistry (C1), Biotechnology (C2) and Pharmaceuticals (C3), Chemistry engineering (CA) and Environmental technology (CB), and so on. In Period 2 such technology convergence networks became more complicated. In the Electrical engineering sector, technologies such as Audio-visual technology (E2), Telecommunications (E3), Digital communication (E4), Computer technology (E6) and IT methods for management (E7) converged with Mechanical elements (M7), Optics (I1), Surface technology, coating (C8), Micro-structural and nano-technology (C9), while technologies in the Chemistry sector such as Organic fine chemistry (C1), Biotechnology (C2) and Pharmaceuticals (C3) developed more additional linkages to technologies in other sectors, *e*.*g*., Analysis of biological materials (I3). In addition to that, we note characteristic technology convergence networks in the industries of the Instruments sector. While Measurement (I2) was the main technology field of convergence in the case of the university or PRI patents, Optics (I1) was in case of the industry patents. Such a difference may be attributed to commercialization of laser processing and optical communication technologies as a result of industrial R&D activities. Further developments in technology convergence networks in Period 3 connect most technology fields one another, as shown in Fig 4(C).

#### Institution-Type-Dependent characteristics of key fields of technology convergence

Figs 2, 3 and 4 show the results of network analysis highlighting the technologies of which convergence frequently occurred. Identification of such key technology fields having higher chances of technology convergence can provide crucial information in strategic managerial decisions on such as corporal decisions in R&D investments and planning of governmental R&D policies.

Table 4 summarizes the top five technology fields in terms of node strength segmented by institution type in either of Period 1, 2 or 3. In other words, Table 4 shows changes over periods in the key fields of node strength for technology convergence according to institution type.

**Table 4:** Top five node strength (in parentheses) of technology fields in technology convergence network by institution type, grouped into three periods.

Institution	Rank	Period 1 (1997–2001)	Period 2 (2002–2006)	Period 3 (2007–2011)
PRI	1	Micro-structural and nano-technology (2.8)	Digital communication (12.5)	Digital communication (53.6)
	2	Electrical machinery, apparatus, energy (2.4)	Micro-structural and nano-technology (11.6)	Measurement (52.9)
	3	Materials, metallurgy (2.4)	Telecommunications (9.4)	Telecommunications (48.2)
	4	Digital communication (2.1)	Computer technology (7.7)	Computer technology (42.3)
	5	Semiconductors (2)	Materials, metallurgy (5.3)	Electrical machinery, apparatus, energy (36.7)
University	1	Micro-structural and nano-technology (1.4)	Micro-structural and nano-technology (14.5)	Biotechnology (74.9)
	2	Semiconductors (0.8)	Materials, metallurgy (4.9)	Measurement (74.8)
	3	Electrical machinery, apparatus, energy (0.5)	Semiconductors (4.1)	Computer technology (74)
	4	Materials, metallurgy (0.5)	Digital communication (4.1)	Micro-structural and nano-technology (69.6)
	5	Chemical engineering (0.4)	Computer technology (3.6)	Electrical machinery, apparatus, energy (66.6)
Industry	1	Digital communication (108)	Digital communication (409.3)	Semiconductors (940.9)
	2	Telecommunications (90.2)	Telecommunications (402.2)	Electrical machinery, apparatus, energy (932)
	3	Computer technology (48.2)	Audio-visual technology(335.8)	Computer technology (795.5)
	4	Audio-visual technology (44.8)	Computer technology (292.1)	Audio-visual technology (795)
	5	Electrical machinery, apparatus, energy (32.7)	Electrical machinery, apparatus, energy (262)	Optics(765.9)

In PRI patents, Micro-structural and nano-technology (C9) earned the top rank in Period 1. However, its ranking fell to the second in Period 2 and even below the top five in Period 3. While Electrical machinery, apparatus, energy (E1) and Materials, metallurgy (C7) show a similar drop in node strength ranking over time, communication, measurement and computer-related technologies such as Telecommunications (E3) and Digital communication (E4) do the other way around, particularly from Period 2.

The node strength rankings of the industry patents over time, however, moved in the opposite way from the PRI patents. We find an instance in Telecommunications (E3) and Digital communication (E4), of which industry patents had one of the highest node strength in Period 1 but had disappeared in Period 3. This may be attributed to that industries had the earlier technology convergence than universities or PRIs. In such a case, technology convergence in PRI patents of Telecommunications (E3) and Digital communication (E4) would hardly play a predominant role in enhancing the standardized communication-related technologies. Meanwhile, the node strength ranking of the PRI Measurement (I2) patents was recently (*i*.*e*., in Period 3) the second highest. Considering the significance of measurement technologies in industry standardization [72,73], this seems to provide another piece of evidence accounting for the role of PRIs in the establishment of industry standards.

Micro-structural and nano-technology (C9) took the top node strength of technology convergence networks in the university patents from Period 1 to Period 2. This is because universities had in general played a pioneering key role in nanotechnology researches, many of which began to find commercial applications after the latter half of Period 2 [74,75]. For instance, development of patterning technologies for miniaturized devices often demands interdisciplinary approaches integrating chemistry, materials science and even mechanical engineering. It was universities that facilitated such convergence of technologies. As industries accomplished the commercialization of miniaturized fabrication, however, the ranking of university technology convergence dropped. The decline in the node strength ranking of Semiconductors (E8) and Materials, metallurgy (C7) can be accounted for in a similar way. Instead, Biotechnology (C2) and Measurement (I2) took the predominance in the node strength ranking of technology convergence in Period 3.

Industry patents show different network characteristics of technology convergence from university or PRI patents. Both Periods 1 and 2 had Digital communication (E4), Telecommunications (E3) and Electrical machinery, apparatus, energy (E1) in the first, second and fifth ranking of node strength, respectively. Computer and Audio-visual technologies took the third and fourth ranks in Period 1, respectively, which was switched each other in Period 2. The top five node strength technologies in Periods 1 and 2 indicate the vigorous convergence of technologies around telecommunications (E3), *e*.*g*., GSM (groupe spécial mobile) and CDMA (code division multiple access) routers and handsets, personal computing and television (TV) sets. On the other hand, in Period 3, Semiconductors (E8) and Optics (I1) appeared in the first and fifth ranking of node strength. Rise of their ranking can be attributed to the diffusion of intensive information and communications technology (ICT)-centric R&D investments backed up by the Korean government, of which applications included smartphones as the core of technology convergence.

In summary, the convergence of technology fields, especially with communication-related ones, in the Electrical engineering sector resulted in a high level of node strength, regardless of institution type until Period 2. When it comes to Period 3, however, only PRI patents kept such a high level of node strength in the sector. High node strength in Audio-visual technology (E2) of industry patents may be attributed to the strength of Korea in consumer electronics including TVs and monitors. Industries also appeared to lead technology convergence with Computer technology (E6), while universities and PRIs followed since Period 2. Similarly, in the case of Electrical machinery, apparatus, energy (E1), high node strength of technology convergence around the patents of PRIs or universities (as early as Period 1) preceded that of industry counterparts (after Period 2). On the other hand, Semiconductors (E8) corresponds to a counter-example of transition in node strength from either of PRIs or universities (in Periods 1 and 2) to industries (in Period 3). Period 3 shows that, in industries, Optics (I1) occupied the top node strength, while Measurement did in universities and the PRIs. In the Chemistry sector, Micro-structural and nano-technology (C9), Materials, metallurgy (C7) and Biotechnology (C2) were among the top five node strength technology fields for convergence, especially for PRIs and universities.

#### Separation of Technology-Convergence fields according to institution type

**Fig 5:**
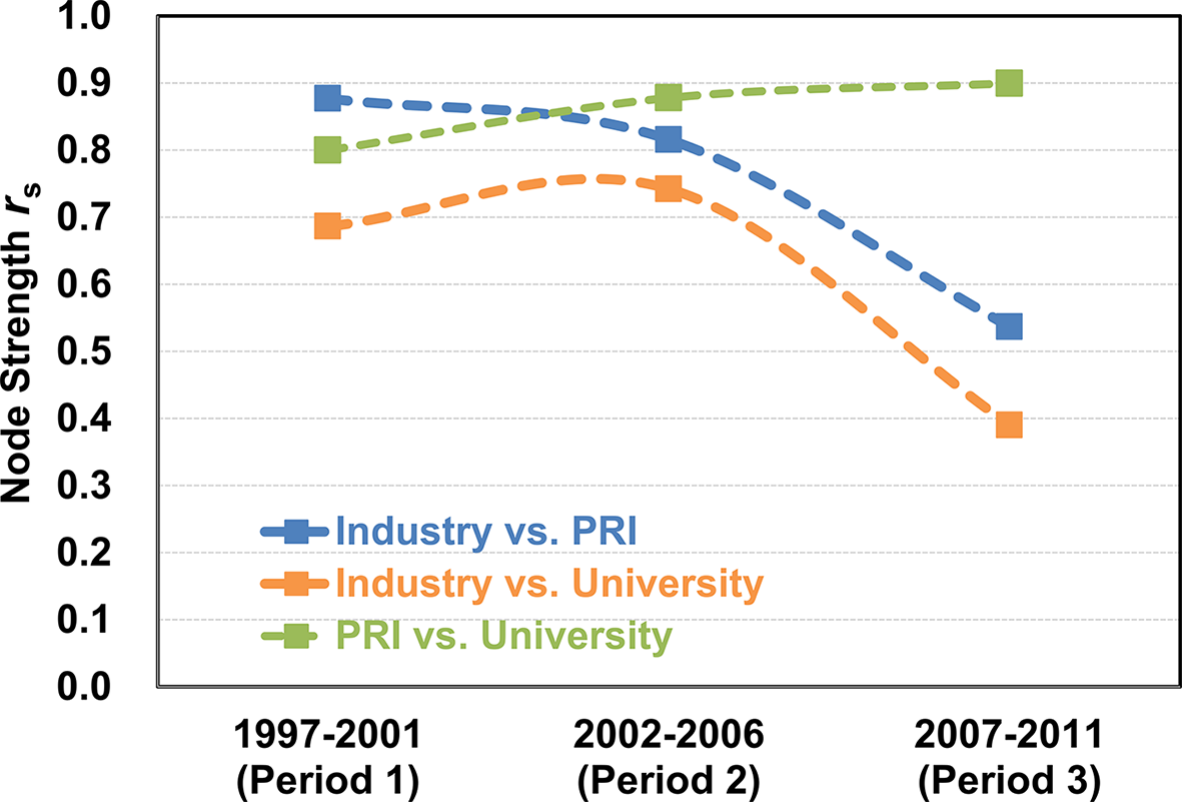
Changes in the correlational characteristics of the technology convergence networks across institution type. The Spearman correlation coefficients (*r*_*s*_) between the types of institutions were calculated using node strength.

Figs 2 to 4 indicate diversification in the evolution of the technology convergence networks according to institution type. To measure how idiosyncratic an institution type has developed a technology convergence network of its own, we analyzed the non-parametric rank order correlation between the networks of one institution type and that of another, using the Spearman correlation coefficient *r*_*s*_ [76]. Fig 5 shows the results of the Spearman correlation analysis, where the *r*_*s*_ was calculated from the node strength of each institution type. All the coefficients were statistically significant: all the coefficients had p value smaller than 0.01 except a single point, in which the p values of *r*_*s*_ between industry and university for Period 3 (2007–2011) from node strength was 0.0205.

Fig 5 shows a piece of evidence for diversification in the technology fields of convergence from one institution type to the others: industry is distinctive in that it has developed virtually its own technology convergence during 2007 to 2011, *i*.*e*. in Period 3. Furthermore, it needs to be noted that the industry had the highest *r*_*s*_ of the technology convergence between industry and PRI, of which decrease in *r*_*s*_ was the most drastic from 0.88 to 0.54; *r*_*s*_ between industry and university also decreased from 0.69 to 0.39. Meanwhile, the PRI and university technology convergence networks became increasingly similar each other, with increase in their *r*_*s*_ between PRI and university from 0.80 to 0.90.

Fig 5 suggests that the technology fields play a significant role in evolution of characteristic technology convergence networks according to institution type. Such a separation in the fields of technology for convergence may be due to specialization in institutional role in R&D [77]. In other words, our findings imply that understanding *both* the institutional and the field-specific characteristics of technology convergence may contribute to more efficient R&D investment or policy decision. Further study on the factors behind the separation of convergent technologies may improve our understanding on innovation.

#### Institution-Type-Dependent trends in technology convergence: Which technologies are combined?

Using various indices we found the most frequent instances of technology convergence during the most recent period, *i*.*e*., Period 3. Table 5 shows the top five frequent combinations of technology fields normalized to the total number of technology-convergent patents. The normalized weight of links of technology convergence was used to relatively compare the number of patents by one institution type to that by another. This is related to the implications of this study, which can be utilized in planning of R&D policies or determination of R&D investment. Table 5 indicates that, regardless of institution type, technology convergence was most frequent around either of Telecommunication and Digital communication (E4), Organic fine chemistry (C1) and Pharmaceuticals (C3). However, further scrutiny reveals that convergence around Telecommunication and Digital communication (E4) occurred more frequently in PRI or industries patents than in university patents.

**Table 5:** Top five combinations of technology fields in technology convergence by institution type, based on the relative fraction, from 2007 to 2011.

Rank	PRI		University		Industry	
	Convergence between	Degree	Convergence between	Degree	Convergence between	Degree
1	Telecommunications + Digital communication	13.6%	Telecommunications + Digital communication	7.0%	Telecommunications + Digital communication	7.0%
2	Measurement + Analysis of biological materials	4.9%	Analysis of biological materials + Biotechnology	5.7%	Organic fine chemistry + Pharmaceuticals	4.4%
3	Digital communication + Computer technology	4.2%	Biotechnology + Pharmaceuticals	4.7%	Audio-visual technology + Optics	3.4%
4	Analysis of biological materials + Biotechnology	3.3%	Organic fine chemistry + Pharmaceuticals	4.7%	Semiconductors + Optics	3.4%
5	Organic fine chemistry + Pharmaceuticals	3.2%	Measurement + Analysis of biological materials	4.6%	Telecommunications + Computer technology	3.2%

On the other hand, Table 5 reconfirms that the main fields of technology convergence vary with institution type, which is similar to the findings of Table 4. For example, neither PRIs nor universities had technology convergence of Audio-visual technology (E2) with Optics (I1, taking the third ranking in the industry patents) and of Semiconductors (E8) with Optics (I1, the fourth in the industry patents) in the top five frequent combinations of technologies. In other words, it was industries that actively carried out the technology convergence with Optics (I1). The opposite happened in the technology convergence of Analysis of biological materials with Measurement (I2) as well as that of Analysis of biological materials with Biotechnology (C2). Because of the fundamental nature of the biology-related technology, universities or PRIs would have played a central role in the relevant R&D.

Table 6 summarizes the top five instances of technology convergence based on the technological closeness of the technology fields. Interestingly, the most frequent combinations of technologies in Table 6 are not identical to that in Table 5, varying according to institution type. This does not necessarily mean that the most frequent combinations of technology fields in Table 6 were completely different from that in Table 5. For instance, convergence of technologies with Analysis of biological materials (I3) in Table 6 appear to be one of top five most frequent combinations only in the university and PRI, which matches the results in Table 5. For example, the technology convergence of Telecommunications (E3) with Digital communication (E4) was the most frequent in PRI patents, which was not the case in industry patents. Moreover, in university patents, such a case of technology convergence was not in the top five technology combinations; instead, Organic fine chemistry (C1) and Pharmaceuticals (C3) appears as the most frequent combination. Their technological closeness in university patents and that in industry counterparts were 19.8% and 21.8%, respectively; in other words, patent applications by either of institutions were technology-convergent as many as about one in five. An analysis of technology convergence based on technological closeness regarding Optics (I1) provides another interesting point of view. Convergence of Optics (I1) with Audio-visual technology (E2) and that with Semiconductors (E8) appeared in the top five rankings of Table 5 but they did not in Table 6. This suggests only a few instances of convergence of Optics (I1) with other technology fields in spite of a large number of patents having combinatorial classifications with Optics (I1) and accounts for the diffuse networks of Optics (I1)-related technology convergence visualized in Fig 4.

**Table 6:** Top five combinations of technology fields in technology convergence network by institution type, based on technological closeness, from 2007 to 2011.

Rank	PRI		University		Industry	
	Convergence between	Degree	Convergence between	Degree	Convergence between	Degree
1	Telecommunications + Digital communication	19.2%	Organic fine chemistry + Pharmaceuticals	19.8%	Organic fine chemistry + Pharmaceuticals	21.8%
2	Organic fine chemistry + Pharmaceuticals	14.0%	Macromolecular chemistry, polymers + Other special machines	14.7%	Telecommunications + Digital communication	17.2%
3	Chemical engineering + Environmental technology	13.1%	Analysis of biological materials + Biotechnology	13.0%	Chemical engineering + Environmental technology	13.5%
4	Analysis of biological materials + Biotechnology	10.8%	Materials, metallurgy + Micro-structural and nano-technology	12.2%	Macromolecular chemistry, polymers + Other special machines	11.7%
5	Measurement + Analysis of biological materials	10.6%	Chemical engineering + Environmental technology	12.0%	Biotechnology + Pharmaceuticals	9.4%

Advantages of per-node analyses using such as technological closeness include disclosure of technology convergence that are not readily identified using quantitative counterparts, *e*.*g*., Chemical engineering (CA) and Environmental technology (CB); Macromolecular chemistry, polymers (C4) and Other special machines (M5); Materials, metallurgy (C7) and Micro-structural and nano-technology (C9). Convergence between Chemical engineering (CA) and Environmental technology (CB) occupied high rankings regardless of institution type. This might be attributed to the small number of the corresponding patents, which is often neglected in quantitative analyses. However, as the per-node analysis using technological closeness results show in Table 6, both technologies are close enough to bring about convergence. Another prominent combination of technology convergence in universities is found between Materials, metallurgy (C7) and Micro-structural and nano-technology (C9). Again, this can be attributed to the basic-research-oriented characteristic of university R&D activities [57,58].

## Conclusions

We investigated the evolution of technology convergence characteristic of the type of R&D entity, *i*.*e*. industry, university and PRI using the patent applications filed to KIPO from 1997 to 2011. Based on the definition of technology convergence as a crossover between different technology fields, we constructed the networks according to institution type with patents as the nodes and instances of technology-convergent patents as the weighted links. Examination of the network evolution shows that the key fields of technology convergence varied by institution type. The network density, representing the variety of technology combinations, was the highest in the industry patents, while the rate of increase in network density was the highest in the university patents. This can be attributed to the relatively high degree of freedom in R&D and to the recent increase in the number of university patents. The recent PRI and university technology convergence networks had high node strength in the technology fields of Micro-structural and nano-technology (C9), Materials, metallurgy (C7), Biotechnology (C2) and Measurement (I2), most of which correspond to the fields of the basic science and technology. On the other hand, the industry counterparts were Audio-visual technology (E2), Computer technology (E6) and Optics (I1), many of which R&D outcomes had already been commercialized. Association of the network characteristics between institution types using the Spearman correlation analysis provided another interesting piece of evidence of technology field specialization. We discovered the separation of the key technology fields with which convergence occurred only in industry, while evolutionary characteristics of the technology convergence networks of university and that of PRI appeared to approach each other asymptotically.

We also identified the frequent combinations of technology convergence introducing technological closeness, which were characteristic of institution type as well. In the case of PRI technology convergence, we found the fastest convergence occurred with communication, chemistry and biotechnology-related technologies. In the university patents, on the other hand, the holistic analysis on the network such as network density and node strength showed that communication- and biology-based technologies were the most frequent instances of technology convergence. Meanwhile, the node analysis based on technological closeness identified the chemistry-related technologies were the key fields of technologies convergence. According to the network analysis, the crucial fields of technology convergence of industries were related to ICT, *e*.*g*., such as a combination between Telecommunication and Digital communication (E4), between Telecommunications (E3) and Computer technology (E6), and between Semiconductors (E8) and Optics (I1). Meanwhile, according to the node analysis, chemistry-related fields were identified as the core technologies of convergence, *e*.*g*., between Biotechnology (C2) and Pharmaceuticals (C3) and that between Organic fine chemistry (C1) and Pharmaceuticals (C3).

Our approaches seem appropriate for analysis of technology convergence R&D: firstly, the current status can be identified conspicuously based on the results of quantitative measurement of the network characteristics; secondly, the core fields of technology convergence and the key combinations of technologies can be identified; and finally, based on the analysis and identification, the selection and concentration of supports for such core technology fields can be accomplished, with considerations on the strengths in the R&D entities.

Findings of this study imply that the commercialization of the technologies developed by universities or PRIs affect the characteristics of technology convergence. Networking with external institutions highlights R&D collaboration of universities or PRIs with industries, *e*.*g*., high-tech ventures or small-sized enterprises to foster technology convergence. Meanwhile, governmental supports can play a catalytic role in stimulating technology convergence networks. On the one hand, universities may pioneer the explorative convergence of technologies primarily at the early stages of research, *e*.*g*. by means of hybridizing distinct disciplines. On the other hand, PRIs can provide interconnections between the fundamental researches of university and the commercialization of industry with technology convergence. In Korea such examples can be found in several industry-academic cooperation foundations and in the university-industry cooperation centers (UICCs), respectively

Care should be taken with the co-classification approach in our study as it is not free from limitations. For example, we did not take the collaboration between different types of institution into account because we concentrated on the role of institution type in technology convergence. There may be chances that patents filed by a single applicant have not necessarily been developed in-house. Synthesis of collaborative R&D and technology convergence may give an invaluable piece of evidence enlightening the characteristics of innovation; further research on inter-institutional technology convergence is forthcoming.

Our study has both managerial and policy implications. This is because the network analysis of technology convergence enables targeting of the key technology fields of convergence that is also characteristic of R&D entity type. A government or enterprise, therefore, can utilize such key fields of technology as a guidance for investment priority with considerations on the type of the institution. In this regard, our approach provides a foundation of institutional facilitation for technology convergence R&D, *e*.*g*., in designing and planning national R&D programs.

## References

[1] Dosi G. Technological paradigms and technological trajectories. Res Policy 1982;11(3): 147–162.

[2] Athreye S, Keeble D. Technological convergence, globalisation and ownership in the UK computer industry. Technovation. 2000;20(5): 227–245.

[3] Fai F, von Tunzelmann N. Industry-specific competencies and converging technological systems: evidence from patents. Structural Change and Economic Dynamics. 2001;12(2): 141–170.

[4] Gambardella A, Giuri P, Luzzi A. The market for patents in Europe. Res Policy. 2007;36(8): 1163–1183.

[5] Hacklin F. Management of convergence in innovation: strategies and capabilities for value creation beyond blurring industry boundaries: Contributions to management science Heidelberg, Germany: Springer-Verlag; 2008.

[6] Jeong SK, Kim JC, Choi JY. Technology convergence: What development stage are we in? Scientometrics. 2015;104: 841–871.

[7] Curran CS, Leker J. Patent indicators for monitoring convergence—examples from NFF and ICT. Technol Forecast Soc Change. 2011;78(2): 256–273.

[8] Lind J. Convergence: History of term usage and lessons for firm strategists. Proceedings of ITS 15th Biennial Conference; 2004 Sep 4–7; Berlin, Germany. Calgary, Canada: International Telecommunications Society (ITS); 2004.

[9] McDowall W. Technology roadmaps for transition management: The case of hydrogen energy. Technol Forecast Soc Change. 2012;79(3): 530–542.

[10] Featherston C, O’Sullivan E. A review of international public sector strategies and roadmaps: a case study in advanced materials Cambridge, UK: University of Cambridge; 2014 Available from: http://www.ifm.eng.cam.ac.uk/uploads/Resources/Featherston__OSullivan_2014_-_A_review_of_international_public_sector_roadmaps-_advanced_materials_full_report.pdf.

[11] Etzkowitz H, Leydesdorf L. The dynamics of innovation: from National Systems and "Mode 2" to a Triple Helix of university-industry-government relations. Res Policy. 2000;29: 109–123.

[12] Balconi M, Breschi S, Lissoni F. Networks of inventors and the role of academia: an exploration of Italian patent data. Res Policy. 2004;33(1): 127–145.

[13] Diaz-Perez C, Arechavala-Vargas R. The Role of Public R&D Laboratories in Innovation Networks: a Comparison between Canada and Mexico. In: Cozzens SE, Harari EB, editors. 2007 Atlanta Conference on Science, Technology and Innovation Policy; 2007 Oct 19–20; Atlanta, GA. Washington, DC: IEEE; 2007. p. 1–8.

[14] National Science Foundation (NSF). Science and Engineering Indicators, National Science Board Subcommittee on Science & Engineering Indicators. Washington DC: U.S. Government Printing Office; 2002.

[15] Rosenberg N. Science and Technology: Which Way does the Causation Run?. Stanford, CA: Stanford University; 2004 [cited 2017 May 18]. Available from: http://crei.cat/wp-content/uploads/2016/08/rosenberg.pdf.

[16] Veugelers R. Collaboration in R&D: An Assessment of Theoretical and Empirical Findings. De Economist. 1998;146(3): 419–443.

[17] Sáez CB, Marco TG, Arribas EH. Collaboration in R&D with universities and research centres: an empirical study of Spanish firms. R&D Management. 2002;32(4): 321–341.

[18] Kim H, Park Y. Structural effects of R&D collaboration network on knowledge diffusion performance. Expert Syst Appl. 2009;36(5): 8986–8992.

[19] Parkhe A, Wasserman S, Ralston DA. New Frontiers in Network Theory Development. Acad Manage Rev. 2006;31(3): 560–568.

[20] Kim MS, Kim C. On A Patent Analysis Method for Technological Convergence. Procedia Soc Behav Sci. 2012;40: 657–663.

[21] Lee WS, Han EJ, Sohn SY. Predicting the pattern of technology convergence using big-data technology on large-scale triadic patents. Technol Forecast Soc Change. 2015;100: 317–329.

[22] Rikkiev A. Technology convergence and intercompany R&D collaboration: Across business ecosystems boundaries. International Journal of Innovation and Technology Management. 2013;10(4): 1350009.

[23] Jeong S. Strategic collaboration of R&D entities for technology convergence: Exploring organizational differences within the triple helix. Journal of Management & Organization. 2014;20(2): 227–249.

[24] Lee H, Zo H. R&D allies: How they impact technology convergence in the area of ICT. Proceedings of 2016 International Conference on Information and Communication Technology Convergence (ICTC); 2016 Oct 19–21; Jeju, South Korea. Piscataway, NJ: The Institute of Electrical and Electronics Engineers (IEEE); 2016. doi: 10.1109/ICTC.2016.7763492.

[25] Haeussler C, Patzelt H, Zahra SA, Strategic Alliances and Product Development in High Technology New Firms: The Moderating Effect of Technological Capabilities. Journal of Business Venturing. 2010;27(2): 217–233.

[26] Newman MEJ. The structure of scientific collaboration networks. Proc Natl Acad Sci U S A. 2001;98(2): 404–409. doi: 10.1073/pnas.98.2.404 11149952 10.1073/pnas.021544898 PMC14598

[27] Lee DH, Seo IW, Choe HC, Kim HD. Collaboration network patterns and research performance: the case of Korean public research institutions. Scientometrics. 2012;91: 925–942.

[28] Farré-Perdiguer M, Sala-Rios M, Torres-Solé T. Network analysis for the study of technological collaboration in spaces for innovation. Science and technology parks and their relationship with the university. International Journal of Educational Technology in Higher Education 2016;13: 8.

[29] Reagans R, Zuckerman EW. Networks, diversity, and productivity: The social capital of corporate R&D teams. Organization Science. 2001;12(4): 502–517.

[30] Padula G. Enhancing the innovation performance of firms by balancing cohesiveness and bridging ties. Long Range Planning. 2008:41(4); 395–419.

[31] Rost K. The strength of strong ties in the creation of innovation. Res Policy. 2011;40: 588–604.

[32] Choi JY, Jeong S, Kim K. A Study on Diffusion Pattern of Technology Convergence: Patent Analysis for Korea. Sustainability. 2015;7: 11546–11569.

[33] Hacklin F, Wallin MW. Convergence and interdisciplinarity in innovation management: a review, critique, and future directions. The Service Industries Journal. 2013;33(7–8):774–788.

[34] Curran CS, Bröring S, Leker J. Anticipating converging industries using publicly available data. Technol Forecast Soc Change. 2010;77(3): 385–395.

[35] Geum Y, Kim C, Lee S, Kim M. Technological Convergence of IT and BT: Evidence from Patent Analysis. ETRI Journal. 2012;34(3): 439–449.

[36] Karvonen M, Lehtovaara M, Kässi T. Build-up of understanding of technological convergence: Evidence from printed Intelligence industry. International Journal of Innovation and Technology Management. 2012;9(3): 1250020-1–1250020-24.

[37] Karvonen M, Kässi T. Patent citations as a tool for analysing the early stages of convergence. Technol Forecast Soc Change. 2013;80(6): 1094–1107.

[38] Xing W, Ye X, Kui L. Measuring convergence of China's ICT industry: An input-output analysis. Telecommunications Policy. 2011;35(4): 301–313.

[39] Roco MC, Bainbridge WS, editors. Converging Technologies for Improving Human Performance. Dortrecht, The Netherlands: Kluwer Academic Publishers; 2002.

[40] Bradley CA. Territorial Intellectual Property Rights in an Age of Globalism. Virginia Journal of International Law. 1997;37: 505–586.

[41] Katila R. Measuring innovation performance. International Journal of Business Performance Measurement. 2000;2: 180–193.

[42] Organisation for Economic Co-operation and Development (OECD). Measuring Innovation: A New Perspective. Paris, France: OECD Publishing; 2010.

[43] Ernst H. Patent information for strategic technology management. World Patent Information. 2003;25(3): 233–242.

[44] Jaffe AB. Technological Opportunity and Spillovers of R & D: Evidence from Firms' Patents, Profits, and Market Value. Am Econ Rev. 1986;76(5): 984–1001.

[45] Schmoch U. Concept of a technology classification for country comparisons. Karlsruhe, Germany: Fraunhofer Institute for Systems and Innovation Research: Final Report to the World Intellectual Property Organization (WIPO). Geneva, Switzerland: WIPO; 2008.

[46] Curran CS. The Anticipation of Converging Industries: A Concept Applied to Nutraceuticals and Functional Foods. London, UK: Springer-Verlag; 2013.

[47] Leydesdorff L, Rafols I. A global map of science based on the ISI subject categories. J Assoc Inf Sci Technol. 2008;60(2): 348–362.

[48] Porter AL, Youtie J. How interdisciplinary is nanotechnology? J Nanopart Res. 2009;11(5): 1023–1041. doi: 10.1007/s11051-009-9607-0 21170124 10.1007/s11051-009-9607-0 PMC2988207

[49] Buter RK, Noyons ECM, van Raan AFJ. Searching for converging research using field to field citations. Scientometrics. 2011;86(2): 325–338. doi: 10.1007/s11192-010-0246-0 21297857 10.1007/s11192-010-0246-0 PMC3016233

[50] WIPO. World Intellectual Property Indicators 2012 Edition. Geneva, Switzerland: WIPO; 2012.

[51] Schoen A, Villard L, Laurens P. The network structure of technological developments; Technological distance as a walk on the technology map. STI 2012: International Conference on Science and Technology Indicators; 2012 Sep 5–8; Montreal, Canada. Berlin, Germany: The European Network of Indicator Designers (ENID); 2012. p.733-742.

[52] Leydesdorff L, Kushnir D, Rafols I. Interactive overlay maps for US patent (USPTO) data based on International Patent Classification (IPC). Scientometrics. 2014;98(3): 1583–1599.

[53] Boyack KW, Klavans R. Measuring science-technology interaction using rare inventor-author names, J Informetr. 2008;2(3): 173–182.

[54] Kay L, Newman N, Youtie J, Porter AL, Rafols I. Patent overlay mapping: Visualizing technological distance. J Assoc Inf Sci Technol. 2014;65(12): 2432–2443.

[55] Barnes JA. Class and committee in a Norwegian island parish. Human Relations. 1954;7: 39–58.

[56] Galaskiewicz J, Wasserman S. Social network analysis: Concepts, methodology, and directions for the 1990s. Sociol Methods Res. 1993; 22(1): 3–22.

[57] Wasserman S, Faust K. Social network analysis: Methods and applications. Cambridge, UK: Cambridge University Press; 1994.

[58] Leydesdorff L, Rafols I. Interactive overlays: A new method for generating global journal maps from Web-of-Science data. J Informetr. 2012;6(2): 318–332.

[59] Moody J. The structure of a social science collaboration network: Disciplinary cohesion from 1963 to 1999. Am Sociol Rev. 2004;69(2): 213–238.

[60] WIPO [Internet]. Geneva, Switzerland: World Intellectual Property Organization (WIPO); c1967-2017 [cited 2017 May 18]. IPC concordance table; [about 2 screens]. Available from: http://www.wipo.int/export/sites/www/ipstats/en/statistics/patents/xls/ipc_technology.xls.

[61] Jaccard P. Lois de distribution florale dans la Zone Alpine. Bulletin de la Société Vaudoise des Sciences Naturelles. 1902;38(144): 67–130. French.

[62] Scott J. Social network analysis—A handbook. 2nd ed. Thousand Oaks, CA: Sage Publications; 2000.

[63] Barrat A, Barthelemy M, Pastor-Satorras R and Vespignani A. The architecture of complex weighted networks. Proc Natl Acad Sci U S A. 2004;101(11): 3747–3752. doi: 10.1073/pnas.0400087101 15007165 10.1073/pnas.0400087101 PMC374315

[64] Freeman LC. Centrality in social networks conceptual clarification. Soc Networks. 1979;1: 215–239.

[65] Lee YJ, Kim SU. Measures to Promote Technology Commercialization at Universities and Government-funded Research Institutes. STEPI Insight. 2013 Nov [cited 2017 May 18]; 1:[about 38 p.] Available from: http://www.stepi.re.kr/app/ePublish/view.jsp?cmsCd=CM0240&categCd=A0508&ntNo=1&sort=PUBDATE&sdt=&edt=&src=&srcTemp=&opt=N&currtPg=1.

[66] Suh J. Empirical Analysis of University Patenting in Korea. KDI Journal of Economic Policy. 2010;32(4): 115–151.

[67] Organisation for Economic Co-operation and Development (OECD). OECD Reviews of Innovation Policy Industry and Technology Policies in Korea, Paris: OECD Publishing; 2014.

[68] Jeong S, Lee S, Kim J, Oh S, Kwak K. Organizational Strategy for Technology Convergence. International Journal of Social, Behavioral, Educational, Economic, Business and Industrial Engineering. 2012;6(8): 1989–1995.

[69] Dang Y, Zhang Y, Fan L, Chen H, Roco MC. Trends in worldwide nanotechnology patent applications: 1991 to 2008. J Nanopart Res. 2010;12: 687–706. doi: 10.1007/s11051-009-9831-7 21170123 10.1007/s11051-009-9831-7 PMC2988206

[70] Peercy PS. The drive to miniaturization. Nature. 2000;406: 1023–1026. doi: 10.1038/35023223 10984060 10.1038/35023223

[71] Nalwa HS. Silicon-Based Material and Devices, Two-Volume Set: Materials and Processing, Properties and Devices. San Diego, CA: Academic Press; 2001.

[72] Wise MN. The Values of Precision. Princeton, NJ: Princeton University Press; 1997.

[73] United Nations Industrial Development Organization (UNIDO). Role of measurement and calibration in the manufacture of products for the global market: A guide for small and medium-sized enterprises, Vienna, Austria: UNIDO; 2006.

[74] Godin B. Measurement and Statistics on Science and Technology: 1920 to the Present. Hove, UK: Psychology Press; 2005.

[75] Wissema JG. Towards the Third Generation University: Managing the University in Transition. Cheltenham, UK: Edward Elgar Publishing; 2009.

[76] Myers JL, Well AD. Research Design and Statistical Analysis. 3rd ed. East Sussex, UK: Routledge; 2010.

[77] Cohen WM, Nelson RR, Walsh JP. Links and Impacts: The Influence of Public Research on Industrial R&D. Manage Sci. 2002;48(1): 1–23.

